# Intraperitoneal pyrophosphate treatment reduces renal calcifications in *Npt2a* null mice

**DOI:** 10.1371/journal.pone.0180098

**Published:** 2017-07-13

**Authors:** Daniel Caballero, Yuwen Li, Jonathan Fetene, Julian Ponsetto, Alyssa Chen, Chuanlong Zhu, Demetrios T. Braddock, Clemens Bergwitz

**Affiliations:** 1 Department of Medicine, Section Endocrinology, Yale University School of Medicine, New Haven, CT, United States of America; 2 Endocrine Unit, Massachusetts General Hospital and Harvard Medical School, Boston, United States of America; 3 Department of Pediatrics, The First Affiliated Hospital, Nanjing Medical University, Nanjing, Jiangsu Province, China; 4 Gastroenterology Unit, Massachusetts General Hospital and Harvard Medical School, Boston, United States of America; 5 Department of Infectious Diseases, The First Affiliated Hospital, Nanjing Medical University, Nanjing, Jiangsu Province, China; 6 Department of Pathology, Yale University School of Medicine, New Haven, CT, United States of America; Max Delbruck Centrum fur Molekulare Medizin Berlin Buch, GERMANY

## Abstract

Mutations in the proximal tubular sodium-dependent phosphate co-transporters *NPT2a* and *NPT2c* have been reported in patients with renal stone disease and nephrocalcinosis, however the relative contribution of genotype, dietary calcium and phosphate, and modifiers of mineralization such as pyrophosphate (PPi) to the formation of renal mineral deposits is unclear. In the present study, we used *Npt2a*^*-/-*^ mice to model the renal calcifications observed in these disorders. We observed elevated urinary excretion of PPi in *Npt2a*^*-/-*^ mice when compared to WT mice. Presence of two hypomorphic *Extracellular nucleotide pyrophosphatase phosphodiesterase 1* (*Enpp1*^*asj/asj*^) alleles decreased urine PPi and worsened renal calcifications in *Npt2a*^*-/-*^ mice. These studies suggest that PPi is a thus far unrecognized factor protecting *Npt2a*^*-/-*^ mice from the development of renal mineral deposits. Consistent with this conclusion, we next showed that renal calcifications in these mice can be reduced by intraperitoneal administration of sodium pyrophosphate. If confirmed in humans, urine PPi could therefore be of interest for developing new strategies to prevent the nephrocalcinosis and nephrolithiasis seen in phosphaturic disorders.

## Introduction

Mutations in the sodium phosphate co-transporters *NPT2a [1–3]* and *NPT2c* [4, 5] have been associated with intraluminal stones (nephrolithiasis) and mineral deposits in the renal parenchyma (nephrocalcinosis) in patients with familial forms of hypophosphatemia. In genome-wide association studies, *NPT2a* has also been associated with nephrolithiasis [6] and altered renal function [7, 8]. With both genetic abnormalities affected individuals show renal phosphate-wasting, high circulating levels of 1,25(OH)_2_D, and absorptive hypercalciuria as a result of increased intestinal uptake of calcium [4, 5, 9, 10], and oral phosphate supplements are currently thought to reduce the risk for renal mineralization by lowering circulating levels of 1,25(OH)_2_D and absorptive hypercalciuria [11]. However, the relative contribution of genotype, dietary calcium and phosphate, and modifiers of mineralization to the formation of renal mineral deposits is unclear. Our recent work suggests that reduced levels of *osteopontin (Opn)*, an extracellular matrix factor affecting binding of phosphate to hydroxyapatite crystals, contribute to the development of nephrocalcinosis in *Npt2a*^*-/-*^ mice [12]. This may be due to the fact that *Npt2a*^*-/-*^ mice respond differently to dietary phosphate when compared to WT mice [13]. Further evaluation in the *Npt2a*^*-/-*^ cohort on different diets suggests that urinary calcium excretion, plasma phosphate, and FGF23 levels appear to be positively correlated to renal mineral deposit formation, while urine phosphate levels and the urine anion gap, an indirect measure of ammonia excretion, appear to be inversely correlated [13]. In addition, local tissue levels of Pi generated by tissue nonspecific alkaline phosphatase (Tnsalp) and ectonucleoside triphosphate diphosphohydrolase 5 (Entpd5) may be important as suggested by decreased skeletal mineralization in the absence of these enzymes [14, 15].

In the present report, we hypothesize that genes involved in the synthesis of pyrophosphate (PPi) in the interstitial matrix may be associated with renal mineralization in these mice [16, 17].

PPi is present in plasma at a concentration of 1–6 μM [18] and in urine levels are around 10 μM [19]. Calcium phosphate stone formers appear to have reduced urinary PPi excretion when compared with control subjects [20–23]. Intravenous ^32^PPi is rapidly hydrolyzed in plasma by *tissue nonspecific alkaline phosphatase (Tnsalp)* that is expressed in the proximal tubules of the kidneys [24] and less than 5% of intravenous ^32^PPi appears in urine. These data indicate that urine PPi is generated locally in the kidneys [25, 26].

*Extracellular nucleotide pyrophosphatase phosphodiesterase 1 (Enpp1)* hydrolyzes extracellular ATP into AMP and PPi and may be an important source of extracellular PPi in the body [27, 28]. *Enpp1* is the founding member of the ENPP or NPP family of enzymes [29]. It has phosphodiesterase activity [27] and is a type II extracellular membrane bound glycoprotein located on the mineral-depositing matrix vesicles of osteoblasts and chondrocytes [30] and the vascular surface of cerebral capillaries [28]. *Enpp1* is also expressed in the kidney collecting duct and possibly other segments [25]. The second source of PPi generation in the kidney is the mevalonate pathway inside mitochondria [26]. Intracellular PPi is released into the interstitium and the urine by the transporter *progressive ankylosis gene product (Ank)* [31]. *Ank* is located at the apical membrane of collecting ducts suggesting that it may function to inhibit mineralization within the tubule lumen. Additionally, *ecto-5-prime nucleotidase (Nt5E/CD73)*, which inhibits *Tnsalp* by further hydrolyzing AMP to adenosine, and *adenosine triphosphate-binding cassette* [32], and *subfamily C*, *member 6 (Abcc 6)*, recently shown to secrete ATP from hepatocytes [32], may both be involved in PPi generation.

In the present study, we used *Npt2a*^*-/-*^ mice to model these disorders. Renal mineral deposits in *Npt2a*^*-/-*^ mice are found at intraluminal and interstitial sites, they contain calcium, phosphorus and osteopontin, and it has been suggested that they ultrastructurally resemble the composition of Randall’s plaques [33, 34]. The extent of renal mineralization is highest between newborn and weaning age *Npt2a*^*-/-*^ mice [35]. Mineralization resolves subsequently on 0.3–0.6% dietary phosphate, but persists beyond weaning age when diets are supplemented with 1.65% phosphate [35] or 1.2% phosphate [12, 36]. Ablation of *25(OH)-vitamin D-1-alpha hydroxylase (Cyp27a1)* prevents renal mineralization, as shown in *Cyp27a1*^*-/-*^*/Npt2a*^*-/-*^ double-knockout mice [35].

We here report that urine PPi levels are increased in *Npt2a*^*-/-*^ mice when compared to WT mice, possibly to protect from renal mineralization in the setting of hyperphosphaturia. Presence of two hypomorphic *Enpp1*^*asj/asj*^ alleles decreases urine PPi and worsens renal calcium phosphate deposit formation in *Npt2a*^*-/-*^ mice. Conversely, development of mineral deposits in these mice can be reduced by intraperitoneal administration of sodium pyrophosphate. These studies suggest that PPi may be a thus far unrecognized factor modulating the development of renal calcifications in *Npt2a*^*-/-*^ mice which may be, if confirmed in humans, of diagnostic and therapeutic relevance for phosphaturic disorders.

## Materials and methods

### Animals

Male and female C57BL/6 mice were obtained from Charles River Laboratory, MA. Male and female *Npt2a*^*-/-*^ mice (B6.129S2-*Slc34a1*^*tm1Hten*^/J, Stock No: 004802), and *Enpp1*^*asj/asj*^ mice (C57BL/6J-*Enpp1*^asj^/GrsrJ, Stock No: 012810) were purchased from The Jackson Laboratory, ME. The *Enpp1*^*asj*^ allele is partially active and shows approximately 15% level of *Enpp1* activity compared to wild-type controls [37]. Mice were genotyped by PCR amplification of genomic DNA extracted from tail clippings as described [29, 38–40]. Mice were weaned at 3 weeks of age and allowed free access to water and regular chow (1.0% calcium, 0.7% phosphorus, of which 0.3% phosphorus is readily available for absorption, Harlan Teklad TD.2018S). Mice received daily intraperitoneal (i.p.) injection of Hanks Buffered Saline (Gibco, Life Sciences) or sodium pyrophosphate in HBSS for two weeks until age four weeks as previously described (160 micromole/Kg/day) according to [41]. To determine whether renal mineral deposits persist beyond weaning age mice were followed for an additional 10 weeks of age after weaning on regular chow. The background of all mouse lines is C57Bl6, use of littermates for controls further reduced bias based on genetic background. No difference in renal mineral deposits was observed between sexes as previously reported by us [12, 36] and thus genders were combined here.

Mice were euthanized following orbital exsanguination in deep anesthesia with isoflurane and vital organs were removed as described [12, 36]. The research under IACUC protocol 2014–11635 was first approved Oct. 22 2014 by the Yale Institutional Animal Care and Use Committee (IACUC), was renewed Sept. 7 2016, and is valid through Sept. 30 2017. Yale University has an approved Animal Welfare Assurance (#A3230-01) on file with the NIH Office of Laboratory Animal Welfare. The Assurance was approved May 5, 2015.

### Blood and urine parameters

Biochemical analyses were done on blood samples (taken by orbital exsanguination) and spot urines collected following an overnight fast at the same time of day between 10 AM and 2 PM. Following deproteinization of heparinized plasma by filtration (NanoSep 300 K, Pall Corp., Ann Arbor, MI), plasma and urinary total pyrophosphate (PPi) concentrations were determined using a fluorometric probe (AB112155, ABCAM, Cambridge, MA). Urine PPi was corrected for urine creatinine, which was measured by LC-MS/MS or by ELISA using appropriate controls to adjust for inter-assay variability.

### Kidney histology

Left kidneys were fixed in 4% formalin/PBS at 4°C for 12 h and then dehydrated with increasing concentration of ethanol and xylene, followed by paraffin embedding. Mineral deposits were determined on 10 um von Kossa stained sections counterstained with 1% methyl green. Hematoxyline/eosin was used as counterstain for morphological evaluation. Histomorphometric evaluation of sagittal kidney sections that includes cortex, medulla and pelvis was performed blinded by two independent observers using an Osteomeasure System (Osteometrics, Atlanta, GA). Percent calcified area was determined using the formula: % calc. area = 100*calcified area/total area (including cortex, medulla and pelvic lumen), and is dependent on number of observed areas per section. Mineralization size was determined using the formula: calc. size = calcified area/number of observed calcified areas per section.

For transmission electron microscopy, a 1 mm^3^ block of the left kidney was fixed in 2.5% glutaraldehyde and 2% paraformaldehyde in phosphate buffered saline for 2 hrs., followed by post-fixation in 1% osmium liquid for 2 hours. Dehydration was carried out using a series of ethanol concentrations (50% to 100%). Renal tissue was embedded in epoxy resin, and polymerization was carried out overnight at 60°C. After preparing a thin section (50 nm), the tissues were double stained with uranium and lead and observed using a Tecnai Biotwin (LaB6, 80 kV) (FEI, Thermo Fisher, Hillsboro, OR) at the Yale Center for Cellular and Molecular Imaging (YCCMI).

### Renal gene expression analysis

Right kidneys were used for preparation of total RNA using Trizol (Thermo Fisher Sci, Inc., Waltham, MA). qRT-PCR (Omniscript, QuantiTect, Qiagen, Valencia, CA) was performed in an ABI-Step One Plus Cycler (Fisher, Life Technologies, Waltham, MA) using the mouse beta actin forward primer: GGCTGTATTCCCCTCCATCG, and reverse primer: CCAGTTGGTAACAATGCCATGT, the mouse *Enpp1* forward primer: CTGGTTTTGTCAGTATGTGTGCT and reverse primer: CTCACCGCACCTGAATTTGTT, the mouse *Entpd5* forward primer: CCAAAGACTCGATCCCCAGAA and reverse primer: TGTTAGAAAGTTCACGGTAACCC, the mouse *Ank* forward primer: TACGGGCTGGCGTATTCTTTG and reverse primer: CACTGTAGGCTATCAGGGTGT, and the mouse *Tnsalp* forward primer CCAACTCTTTTGTGCCAGAGA and reverse primer: GGCTACATTGGTGTTGAGCTTTT.

### Statistical analysis

Data are expressed as means±SEM and analyzed in Microsoft Excel 2010 or Graphpad Prism 6.0. Differences were considered significant if p-values, calculated using the unpaired, two-tailed Student’s t-test, linear regression analysis, or one-way ANOVA using Tukey’s adjustment for multiple comparisons, were smaller than 0.05.

## Results

### Renal PPi excretion is increased *Npt2a*^-/-^ mice

Humans with loss-of-function of *NPT2a [1–3]* and *NPT2c* [4, 5] develop renal mineralization, which may manifest during early childhood prior to specific therapy or when inappropriately receiving active vitamin D analogs, but can also occur throughout life [9]. To model these kidney abnormalities, we used 2 months old *Npt2a*^*-/-*^ mice [39, 40] placed on a diet containing 0.6% calcium and 0.7% phosphorus (Harlan Teklad TD.2018S).

**Fig 1:**
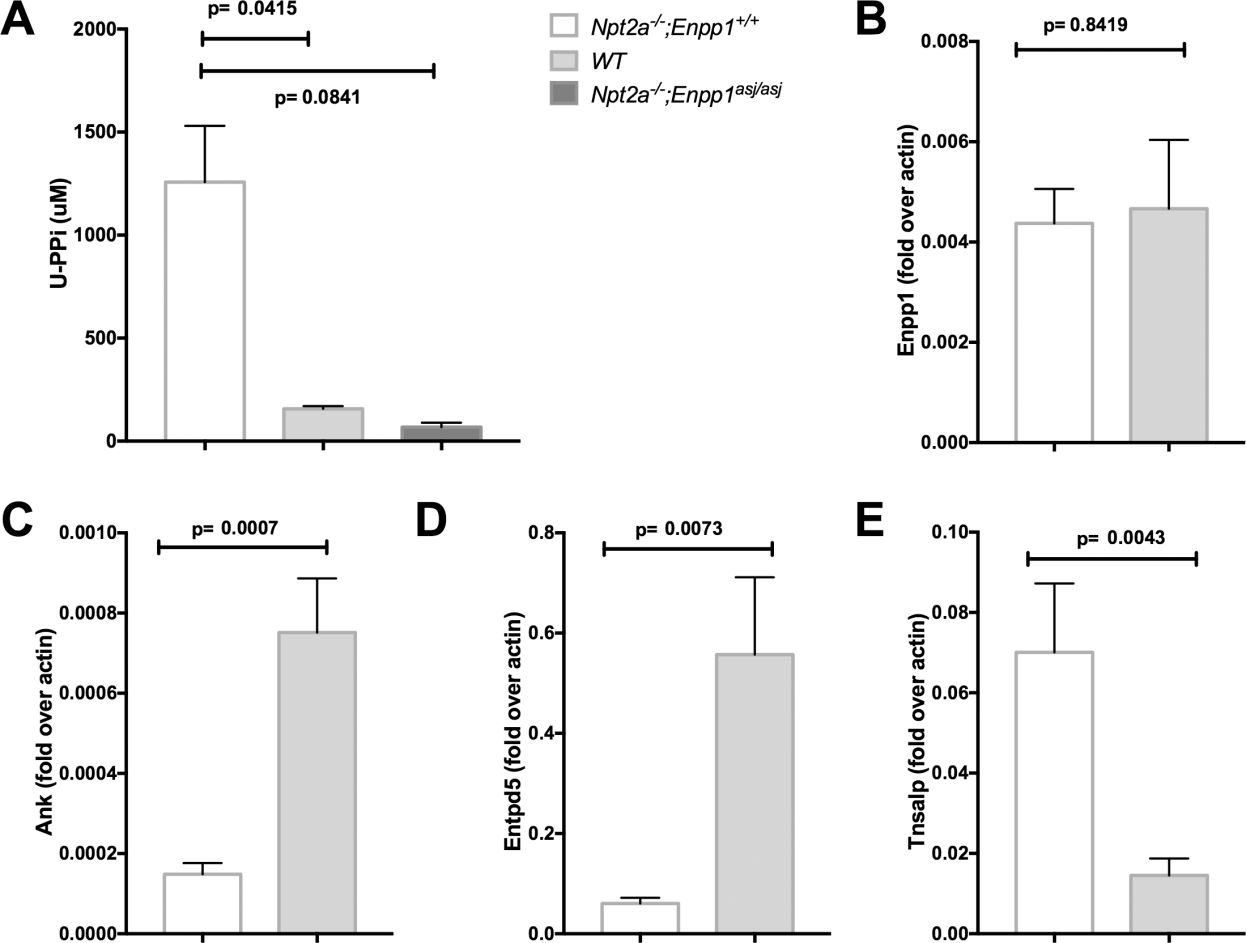
Urine PPi concentration and renal gene expression in *Npt2a*^*-/-*^ mice. Urine pyrophosphate concentration (U-PPi, **A**) following an overnight fast and renal gene expression as indicated on the y-axis for *ectonucleotide pyrophosphatase/phosphodiesterase 1* (*Enpp1*, **B**), *progressive ankylosis* (*Ank*, **C**), *ectonucleoside triphosphate diphosphohydrolase 5* (*Entpd5*, **D**), *tissue nonspecific alkaline phosphatase* (*Tnsalp*, **E***)* in mice fed regular chow for 10 weeks. The data represent mean±SEM of 4–19 mice, p-values shown above the lines of comparisons were calculated by one-way ANOVA using Tukey’s adjustment for multiple comparisons (**A**) and Student’s t-test (**B-E**).

Interestingly, the urine PPi concentration was increased in *Npt2a*^*-/-*^ mice (1257±272 micromole/l, n = 19 vs. WT 157±13 micromole/l, n = 7, p = 0.042) (Fig 1A).

Similarly, urine PPi excretion corrected for urine creatinine was increased in *Npt2a*^*-/-*^ mice (3.0±0.53 micromole/mg, n = 19 vs. WT 1.3±0.42 micromole/mg, n = 9, p = 0.038) (Panel A in S1 Fig). Evaluation of whole kidney gene expression was unchanged for the PPi-generating enzyme *Enpp1* (0.004±0.001, n = 9 vs. WT 0.005±0.001, n = 7, p = ns) and decreased for the PPi transporter *Ank* (0.00015±2.8e-5, n = 9 vs. WT 0.001±0.00014, n = 10, p = 0.007) (Fig 1B and 1C). Expression of the Pi-generating enzyme *Entpd5* was decreased (0.06±0.01, n = 9 vs. WT 0.6±0.15, n = 10, p = 0.0073) and expression of *Tnsalp*, which hydrolyses PPi to Pi, was increased (0.07±0.02, n = 9 vs. WT 0.02±0.004, n = 10, p = 0.0043) (Fig 1D and 1E). Thus, the source of urine PPi in *Npt2a*^*-/-*^ mice remains unclear and may be extrarenal, localized to a specific tubular segment inside the kidneys, or regulation may occur on the post-translational level.

To further evaluate the role of PPi in renal mineral deposit formation in the setting of renal phosphate wasting we next reduced endogenous PPi production using the hypomorphic murine *Enpp1*^*asj*^ allele [37] or administered sodium pyrophosphate by intraperitoneal injection as previously described [41] to increase PPi.

### Presence of the hypomorphic *Enpp1*^*asj*^ allele blunts urine PPi excretion and worsens renal mineralization in *Npt2a*^*-/-*^ mice

**Fig 2:**
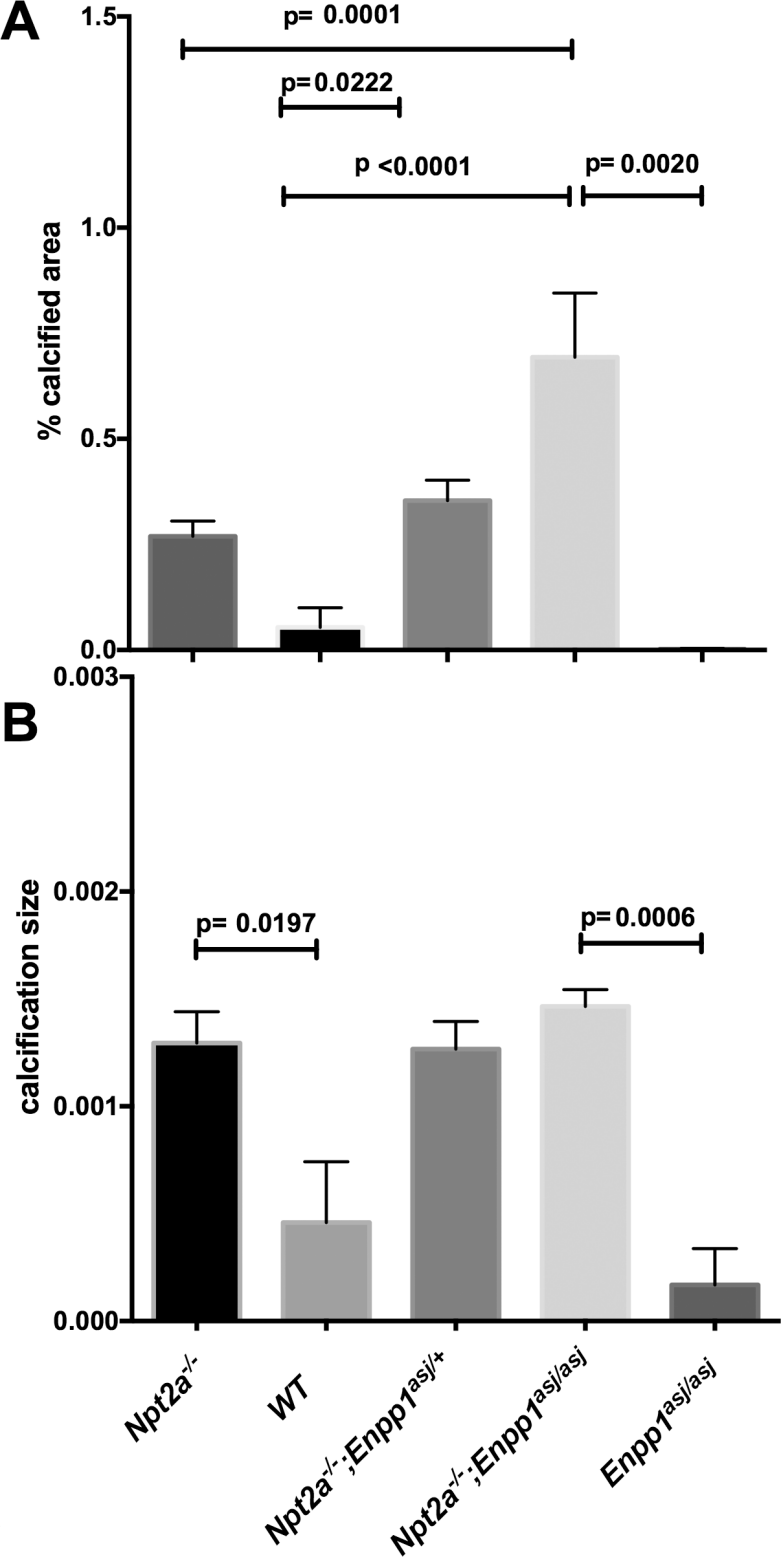
The hypomorphic *Enpp1*^*asj*^ allele worsens renal mineralization area seen in *Npt2a*^*-/-*^ mice on regular chow. Histomorphometric analysis of renal mineralization (%calcified area = 100*mineralization area/tissue area, **A**; calcification size = mineralization area/number of calcifications, um^2^, **B**) in 10 um sections of kidneys from mice fed regular chow for 10 weeks. The data represent individual animals (closed circles) with the means±SEM, p-values shown above the lines of comparisons were calculated by one-way ANOVA using Tukey’s adjustment for multiple comparisons, no significant differences were detected between groups in panel **B**.

**Fig 3:**
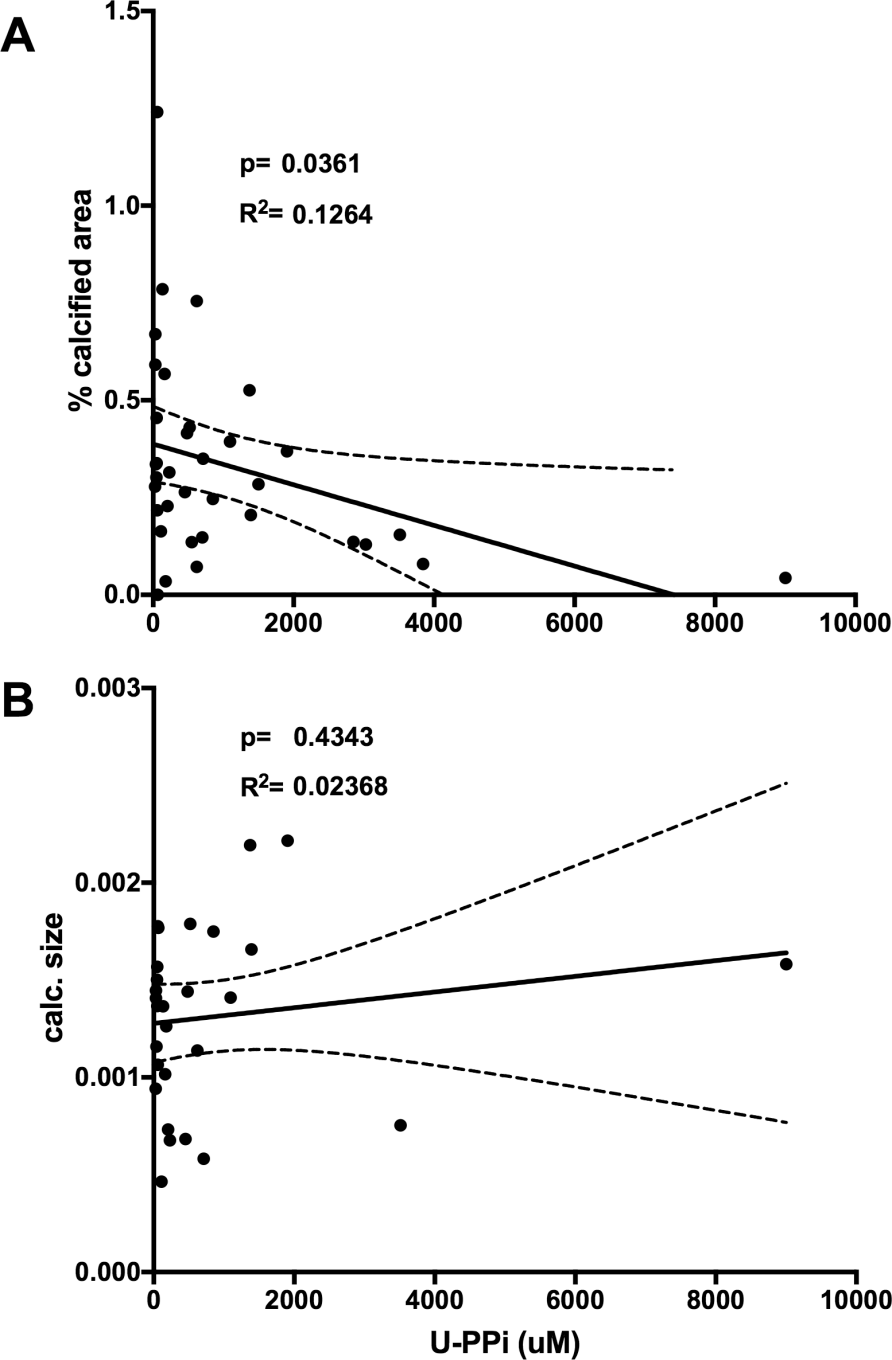
Urinary pyrophosphate concentration is inversely associated with renal mineralization size in a combined bivariate linear regression analysis of all mice. All experimental WT and mutant mice from Fig 2 (n = 28) for which urine was available were evaluated using linear regression analysis to determine the association of renal mineralization with the urine pyrophosphate concentration (U-PPi) (% calcified area = 100*calcified area/total area **A** and calcification size = calcified area/number of mineralization **B**). Data points represent values of individual animals. Results of the linear regression analysis are shown as solid line with 95% confidence interval (stippled lines), R^2^ and p-values.

*Enpp1*^*asj/asj*^ mice develop renal mineralization on a “stone-forming” high phosphorus, low magnesium diet, while they develop no renal mineralization on regular chow [17, 42]. Presence of two hypomorphic *asj* alleles of *Enpp1* blunted the increase of the urine PPi concentration of double-mutant mice when compared to *Npt2a*^*-/-*^ mice on regular chow, albeit non-significantly (67±21, n = 4 vs. *Npt2a*^*-/-*^ 1257±272 micromole/l, n = 19, p = 0.084, Fig 1A). Similarly, urine PPi excretion corrected for urine creatinine was decreased in double mutant mice (0.43±0.084 micromole/mg, n = 4 vs. *Npt2a*^*-/-*^ 3.0±0.53 micromole/mg, n = 19, p = 0.044, panel A in S1 Fig). One or two hypomorphic *asj* alleles of *Enpp1* furthermore increased the calcified area of double-mutant mice when compared to *Npt2a*^*-/-*^ mice on regular chow in a gene dose-dependent fashion (0.3±0.07, n = 8 in *Enpp1*^*asj/+*^*/Npt2a*^*-/-*^, p = ns vs. 0.26±0.04% in *Npt2a*^*-/-*^ and 0.69±0.15% in *Enpp1*^*asj/asj*^*/Npt2a*^*-/-*^, p<0.0001 vs. *Npt2a*^*-/-*^) while no mineral deposits were found in *Enpp1*^*asj/asj*^ mice on regular chow (Fig 2A). Since increased calcified area in double mutants was due to an increase in number of calcifications, no difference was observed for mineralization size between *Npt2a*^*-/-*^, *Enpp1*^*asj/+*^*/Npt2a*^*-/-*^, and *Enpp1*^*asj/asj*^*/Npt2a*^*-/-*^ mice (Fig 2B). Renal calcified area inversely correlated with spot urine PPi concentration (slope = -5.226e-005 ± 2.391e-005, R^2^ = 0.126, p = 0.036) (Fig 3A). No significant correlation was found for calcification area (Fig 3B) or when area and size were correlated with urine PPi corrected for urine creatinine (Panels C and D in S1 Fig).

### Intraperitoneal sodium PPi injection decreases renal mineral deposits in *Npt2a*^*-/-*^ mice

**Fig 4:**
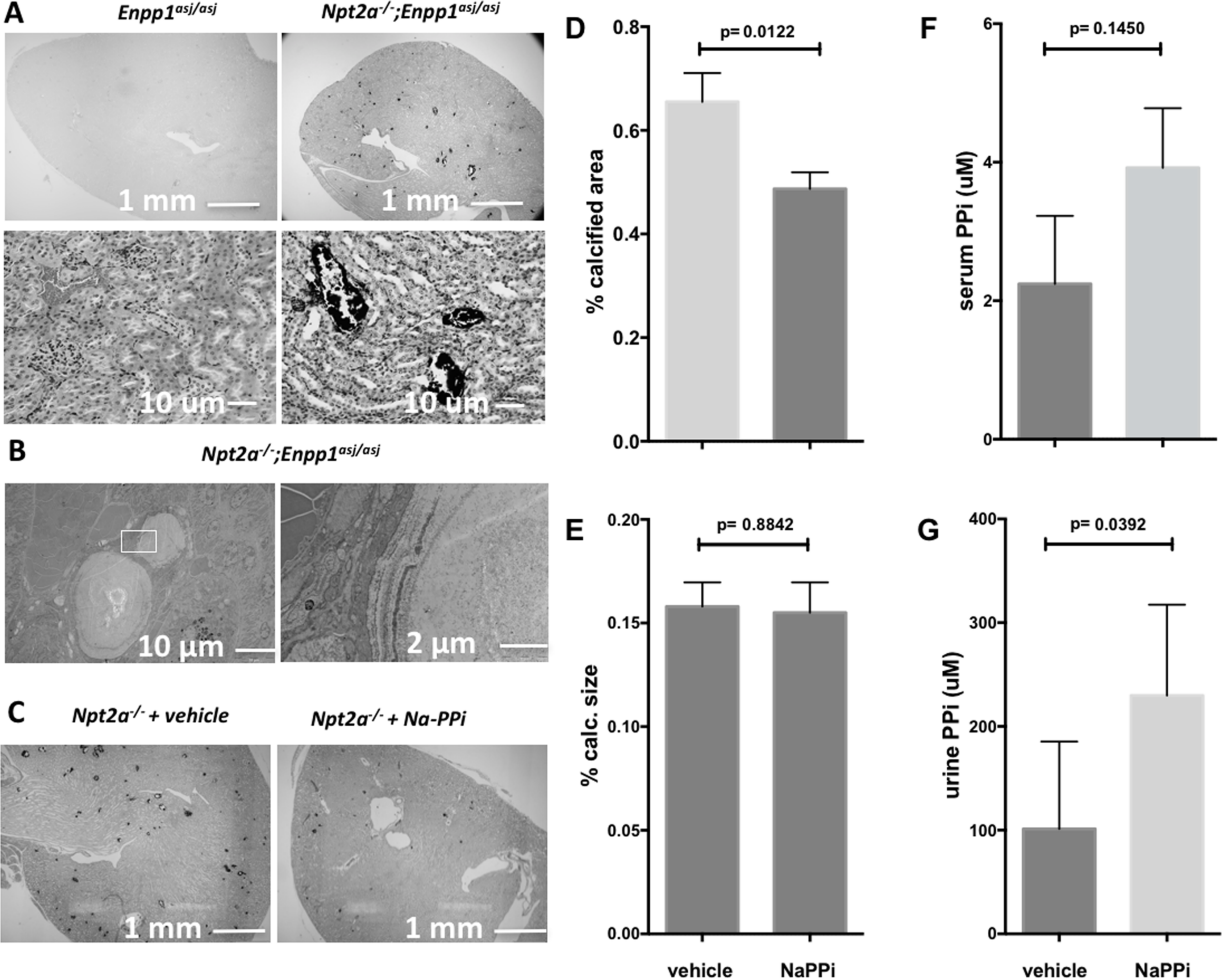
Intraperitoneal injection of Na-pyrophosphate reduces cortical and medulary renal mineralization in *Npt2a*^*-/-*^ mice. Light micrographs of 10 um renal sections prepared from paraffin-embedded kidneys, obtained from mice with various genotypes fed regular chow for 10 weeks (**A, upper panels:** von Kossa, methylene green staining, 4X, and **A, lower panels:** von Kossa, hematoxylin and eosin staining, 40X); Transmission electron micrographs showing microspheres in double mutant mice on regular chow, inset with larger magnification shown to the right (**B**); Two weeks old *Npt2a*^*-/-*^ pups treated with i.p. injections of vehicle or sodium pyrophosphate (160 micromole/Kg/day) for two weeks (**C**); Histomorphometric analysis of renal mineralization (%calcified area = 100*mineralization area/tissue area, (**D)**; calcification size = mineralization area/number of calcifications, um^2^, (**E**), and plasma pyrophosphate levels (**F**) and urine pyrophosphate (U-PPi) (**G**) of two weeks old *Npt2a*^*-/-*^ pups treated with i.p. injections of vehicle or sodium pyrophosphate (160 micromole/Kg/day) for two weeks, measured after overnight fast and 18–24 hrs. following the last treatment. The data represent individual animals (closed circles) with the means±SEM, p-values shown above the lines of comparisons were calculated by Student’s t-test.

Intraperitoneal injection of sodium pyrophosphate was previously shown to reduce arterial calcification in an uremic mouse model [41]. We used the dose of 160 micromole/Kg/day published by these authors and two weeks old *Npt2a*^*-/-*^ pups for this experiment, because renal calcification is more pronounced when compared to older mice (Fig 4A and 4C). Size and body weight (BW) of mice in the treatment group were indistinguishable from vehicle and the animals appeared to be thriving well. Following sacrifice at four weeks of age we observed a reduction of renal mineral deposits by 33% in the treatment group (0.4±0.04, n = 9 vs. vehicle 0.7±0.06%, n = 12, p = 0.01) (Fig 4C and 4D) while mineralization size again was unaffected (Fig 4E). Plasma PPi levels at sacrifice were increased, albeit non-significantly (3.9±0.8, n = 9 vs. vehicle 2.0±0.4 micromole/l, n = 5, p = ns) (Fig 4F). Likewise, the U-PPi concentration was increased (244.9±33.2, n = 14 vs. vehicle 149.4 ± 28.8 micromole/l, n = 14, p = 0.039) (Fig 4G and panel B in S1 Fig).

Histological evaluation showed large interstitial mineral deposits that displaced the surrounding renal tubules. In addition, we observed small intraluminal mineral deposits in cortical and medullary tubular segments of the kidneys of *Npt2a*^*-/-*^ and double-mutant mice (Fig 4A). Transmission electron images showed concentric spheres of similar morphology in *Npt2a*^*-/-*^ and double-knockout mice (Fig 4B) as previously described for *Npt2a*^*-/-*^ mice by us [13, 43] and others [33, 34]. No mineralization was observed in renal vasculature or in the renal pelvis of our mice.

## Discussion

Oral phosphate supplements are currently thought to be the primary intervention to reduce risk for renal mineralization in human carriers of *NPT2a* and *NPT2c* mutations. However, there is concern that oral phosphate therapy might contribute to the formation of renal mineralization despite reduced 1,25(OH)_2_D levels and reduced urinary calcium excretion under certain conditions, for example in patients with X-linked hypophosphatemia (XLH) treated with oral phosphate supplements given multiple times throughout the day [44, 45] and in otherwise healthy individuals following treatment with phosphate enema [46].

We recently reported that reduced urine levels of *osteopontin (Opn)*, an extracellular matrix factor affecting binding of phosphate to hydroxyapatite crystals, contribute to the development of nephrocalcinosis in *Npt2a*^*-/-*^ mice [12]. The present report describes that the urine PPi concentration may be an additional modifier of renal calcifications in this mouse model.

Reduced *Enpp1* activity increased the % calcified area in double mutant mice when compared to *Npt2a*^*-/-*^ mice (Fig 4A), while the size of the calcium phosphate deposits was not affected. Similarly, intraperitoneal sodium PPi treatment reduced % calcified area, while calcification size was unchanged. Although further studies are required to define cause and effect, these data suggest that PPi inhibits nucleation (Figs 2A and 4A), which is different from the effect of *Opn* reported by us [12], that predominantly decreases mineralization size, consistent with the known role of *Opn* in calcium phosphate crystal growth. Interventions that increase both PPi and *Opn* would therefore be predicted to be additive.

*Enpp1* expression is positively regulated by phosphate in osteoblast cultures [47], and therefore we expected that expression is likewise increased in *Npt2a*^*-/-*^ mice to explain the increased urine PPi levels. Instead, we found that *Enpp1* expression is unchanged, possibly as a result of reduced Pi sensing in the absence of *Npt2a*. Furthermore, *Ank* expression was decreased and *Tnsalp* was increased, all predicted to reduce local PPi production. These findings suggest that PPi may be generated outside of the kidneys contrary to previous reports [25, 26], and elevate urine PPi despite unchanged or decreased local gene expression for *Enpp1* and *Ank*, respectively. Consistent with this hypothesis is our finding that global reduction of *Enpp1* activity in *Enpp1*^*asj/asj*^ mutant mice decreased urine PPi levels and that intraperitoneal injection of sodium pyrophosphate increased urine PPi levels (Fig 4G). Alternatively, PPi production may be regulated locally by increased renal activities of *Enpp1* and *Ank* on a post-transcriptional level.

Interestingly, urine PPi in 10 weeks old Npt2a-/- mice is higher than in 4 weeks old weanlings (1257±272 micromole/l vs. 149.4 ± 28.8 micromole/l). This may be a developmental change of urine PPi over the first 10 weeks of life and could be a contributing factor explaining the initial observation in *Npt2a*^*-/-*^ mice reported by the Tenenhouse lab [33], that renal calcifications peak with weaning age and subsequently decrease during adult life in these mice.

Tissue specific ablation of *Enpp1* (and possibly *Ank*) could help determine in future studies whether PPi is produced renally or extrarenally. Injection of recombinant *Enpp1* may be able to reduce the renal calcifications in *Npt2a*^*-/-*^ mice [26, 29] and provide further evidence of the causal relationship of this extracellular enzyme, urine PPi, and renal mineralization.

Also, separate evaluation of interstitial and luminal mineralization and PPi levels and/or activity of PPi generating enzymes may be of interest in future studies. Finally, determining how urinary pH, anion gap, citrate, oxalate, magnesium, and the expression of *uromodulin (Tamm-Horsfall protein*, *THP)* or *Opn* [48] modify PPi action may help better understanding the pathogenesis of renal mineralization in *Npt2a*^*-/-*^ mice.

In summary, we show here that urine PPi is increased in *Npt2a*^*-/-*^ mice. Presence of one or two hypomorphic *Enpp1*^*asj*^ alleles decreases urine PPi and increases renal mineral deposits in *Npt2a*^*-/-*^ mice. Furthermore, the development of nephrocalcinosis and nephrolithiasis in these mice can be reduced by intraperitoneal administration of sodium pyrophosphate. These studies suggest that PPi may be a thus far unrecognized factor modulating the development of renal calcifications in *Npt2a*^*-/-*^ mice which may be, if confirmed in humans, of diagnostic and therapeutic relevance for phosphaturic disorders.

## Supporting information

S1 Fig U-PPi corrected by U-creatinine. Urine pyrophosphate excretion of mice fed regular chow for 10 weeks (U-PPi/U-crea, **A**) and urine pyrophosphate excretion (U-PPi/U-crea) of two weeks old *Npt2a*^*-/-*^ pups treated with i.p. injections of vehicle or sodium pyrophosphate (160 micromole/Kg/day) for two weeks (**B**), measured after overnight fast and 18–24 hrs. following the last treatment. Linear regression analysis to determine the association of renal mineralization with the ratio of urine pyrophosphate/urine creatinine (U-PPi/U-crea) (% calcified area = 100*calcified area/total area **C** and calcification size = calcified area/number of mineralization **D**). The data represent individual animals (closed circles) or means±SEM, p-values shown above the lines of comparisons were calculated by one-way ANOVA using Tukey’s adjustment for multiple comparisons (**A**) and Student’s t-test (**B-D**).
(TIFF)
