# Supplementary figures and images for: Intraperitoneal pyrophosphate treatment reduces renal calcifications in Npt2a null mice
Source: PLoS One. 2017 Jul 13;12(7):e0180098. doi: 10.1371/journal.pone.0180098 (PMC5509111; doi:10.1371/journal.pone.0180098)

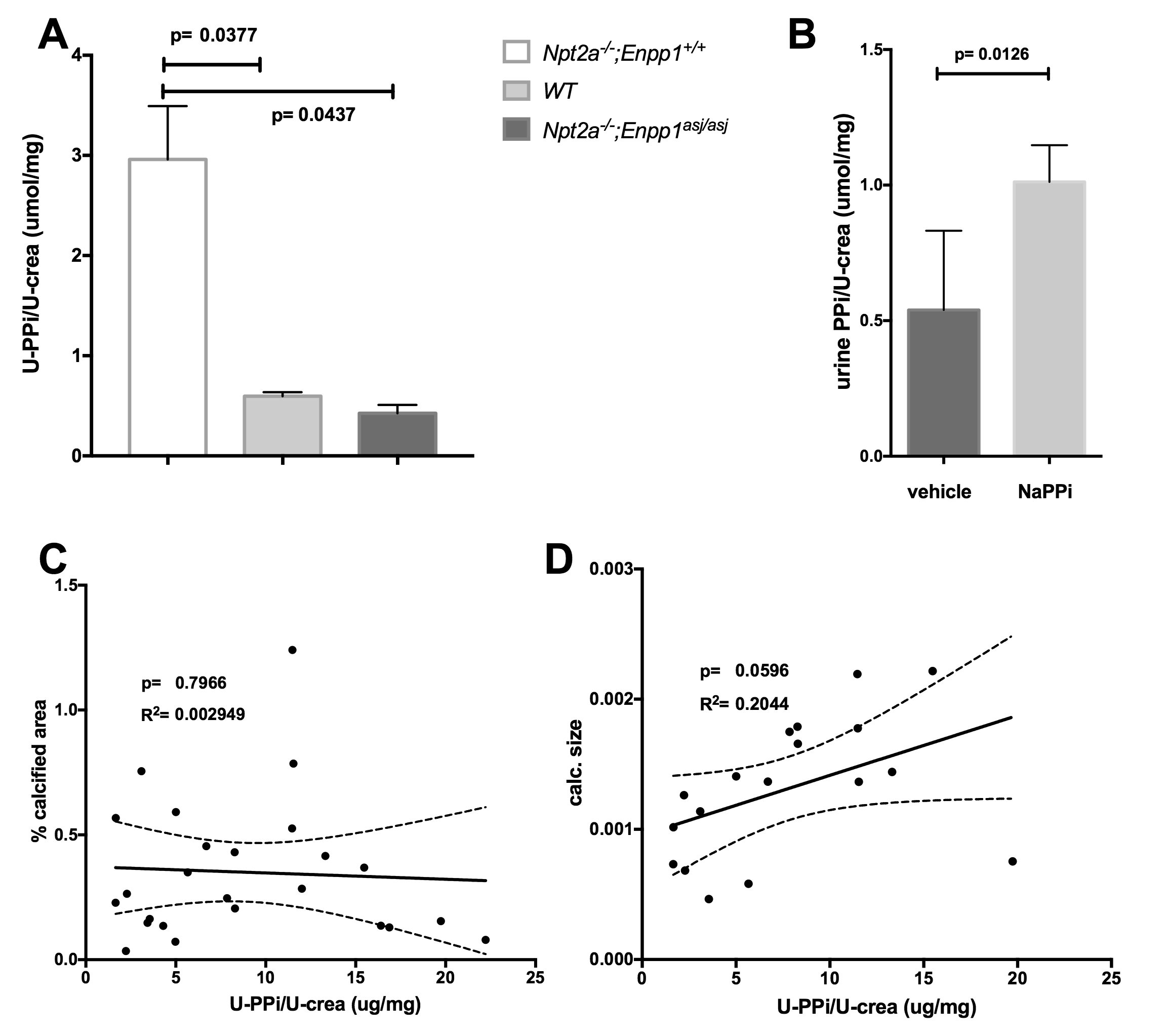

Supplement: S1 Fig — Urine pyrophosphate excretion of mice fed regular chow for 10 weeks (U-PPi/U-crea, A) and urine pyrophosphate excretion (U-PPi/U-crea) of two weeks old Npt2a-/- pups treated with i.p. injections of vehicle or sodium pyrophosphate (160 micromole/Kg/day) for two weeks (B), measured after overnight fast and 18–24 hrs. following the last treatment. Linear regression analysis to determine the association of renal mineralization with the ratio of urine pyrophosphate/urine creatinine (U-PPi/U-crea) (% calcified area = 100*calcified area/total area C and calcification size = calcified area/number of mineralization D). The data represent individual animals (closed circles) or means±SEM, p-values shown above the lines of comparisons were calculated by one-way ANOVA using Tukey’s adjustment for multiple comparisons (A) and Student’s t-test (B-D). (TIFF) [file pone.0180098.s001.tiff]
